# Genome-wide association study of right-sided colonic diverticulosis in a Korean population

**DOI:** 10.1038/s41598-019-43692-8

**Published:** 2019-05-14

**Authors:** Eun Kyung Choe, Jong-Eun Lee, Su Jin Chung, Sun Young Yang, Young Sun Kim, Eun-Soon Shin, Seung Ho Choi, Jung Ho Bae

**Affiliations:** 1 Department of Surgery, Seoul National University Hospital Healthcare System Gangnam Center, Seoul, Korea; 2 DNA Link, Inc., Seoul, Korea; 3 Department of Internal Medicine, Seoul National University Hospital Healthcare System Gangnam, Seoul, Korea

**Keywords:** Genetic association study, Colonic diseases

## Abstract

Diverticulosis results from complex interactions related to aging, environmental factors and genetic predisposition. Despite epidemiologic evidence of genetic risk factors, there has been no attempt to identify genes that confer susceptibility to colonic diverticulosis. We performed the first genome-wide association study (GWAS) on susceptibility to diverticulosis in a Korean population. A GWAS was carried out in 7,948 healthy individuals: 893 patients and 1,075 controls comprised the test set, and 346 patients and 305 controls comprised the replication set. Diverticulosis was diagnosed by colonoscopy during comprehensive medical check-ups, and single-nucleotide polymorphisms (SNPs) related to diverticulosis were detected with the Affymetrix Axiom KORV1.1-96 Array. In all, 9 SNPs were identified in three SNP aggregates in the test set (P < 10^−3^, within 200 kb) after adjusting for sex. All the SNPs were replicated in the replication set (P < 0.05). Three SNPs were near the WNT4 gene, four near the RHOU gene, and two in the OAS1/3 genes. The top SNP associated with right-sided colonic diverticulosis was rs22538787, located near the WNT4 gene [combined set, P-value = 3.128 × 10^−6^, odds ratio = 1.415 (95% confidence interval: 1.223–1.637)]. These 9 novel SNP alleles associated with the WNT4, RHOU, and OAS1/3 genes are possibly involved in the underlying genetic susceptibility to right-sided diverticulosis. Our results provide basic knowledge about the development of diverticulosis in an Asian population.

## Introduction

Colonic diverticulosis is an anatomical alteration characterized by the presence of a hernial sac protruding through a weak area of the intestinal muscle^1^. Although most people with colonic diverticulosis are asymptomatic, approximately 20% of patients develop diverticular disease, and of these, 15% ultimately develop complicated diverticulitis such as colonic perforation, abscess and obstruction during their lifetime^2^. Furthermore, recent knowledge has changed the paradigm of diverticulosis as a chronic bowel disorder that shares common features with irritable bowel syndrome and inflammatory bowel disease^3,4^. In Western and industrialized countries, diverticular disease imposes a significant socioeconomic burden^5^.

The pathogenesis of diverticulosis is not yet fully understood. Traditionally, the formation of colonic diverticula was thought to be due to increased intra-colonic pressure associated with environmental factors such as a Westernized lifestyle and low intake of dietary fiber^6,7^. However, recent epidemiologic data suggest that genetic factors contribute considerably to the occurrence of diverticulosis^8,9^. Recent large twin studies have provided conclusive evidence that genetic factors contribute to the occurrence of diverticulosis and have found that genetic predisposition accounts for approximately 40% to 50% of diverticulosis^8,10,11^. The possibility of genetic contributions to the development of diverticulosis is also supported by interesting observations about differences in anatomic location and prevalence according to ethnicity. Diverticulosis in Western countries is mainly localized to the left side of the colon, and incidence increases with increasing age; however, in Asian countries, including those of Mongolian ancestry, diverticulosis occurs predominantly in the right side of the colon and at a young age^8,12^. The differences in location and prevalence are sustained even after exposure to new environmental factors^13,14^. Moreover, several well-known genetic connective tissue diseases such as Marfan syndrome, Ehlers-Danlos syndrome and polycystic kidney disease have been associated with a higher incidence of diverticulosis^8,9^. The connection between these inherited syndromes and diverticulosis provides strong evidence of a genetic predisposition for diverticulosis and might offer information about its pathogenesis.

Despite this plausible epidemiologic evidence of genetic risk factors, there has been no attempt made to identity genes that confer susceptibility to colonic diverticulosis. Therefore, we report the results of the first genome-wide association study (GWAS) on susceptibility to diverticulosis. The aim of this study was to identify single-nucleotide polymorphisms (SNPs) that could cause right-sided diverticulosis in a Korean population.

## Material and Methods

### Study subjects

**Figure 1:**
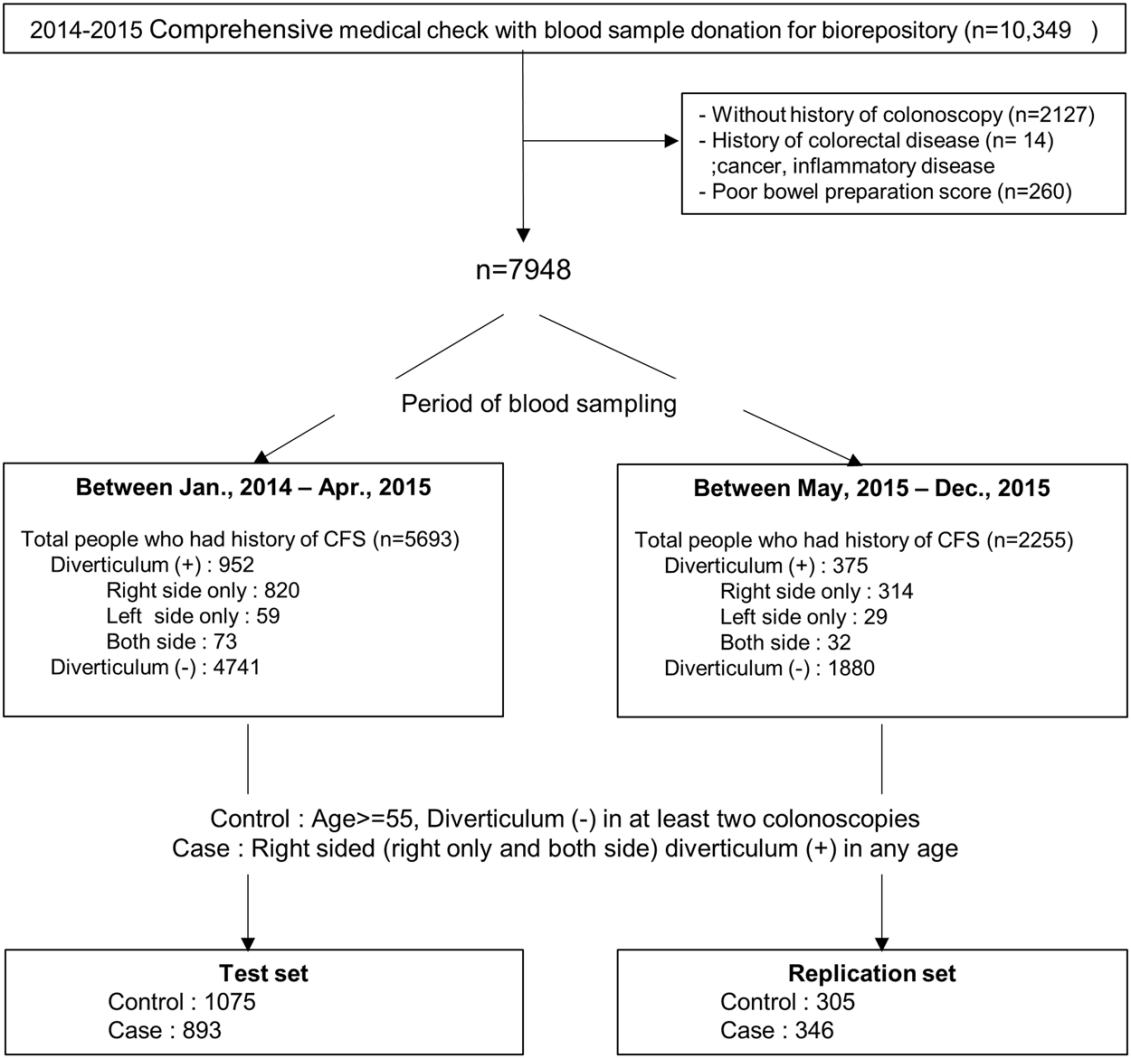
Flow chart of enrollment process.

From 2014 to 2015, 10,349 individuals donated blood samples to the biorepository while participating in a routine comprehensive health check-up program at the Seoul National University Hospital Gangnam Center, after providing informed consent. DNA samples were isolated from the peripheral blood of participants. SNP genotyping was performed by the Hybridization on Affymetrix Axiom KORV1.0-96 Array (Thermo Fisher Scientific, Santa Clara, CA, USA), and the results were stored in the gene-environmental interaction and phenotype database. From this database, we retrospectively collected the data of those who had received a colonoscopy either during the same visit as the blood collection or during a prior visit. A total of 7,948 people remained after applying the following exclusion criteria (Fig. 1): no record of a colonoscopy (n = 2127); incomplete bowel preparation for the colonoscopy (n = 260); or a history of colorectal disease including cancer and inflammatory disease (n = 14).

### Laboratory methods and genotyping

All equipment and resources required for the Axiom 2.0 Assay with automated target preparation are indicated in the Axiom 2.0 Assay Automated Workflow User Guide (P/N 702963, http://www.thermofisher.com/kr/ko/home.html). Using the Axiom 2.0 Reagent Kit (96 reaction, P/N 901758), approximately 200 ng of genomic DNA was amplified and randomly fragmented into 25 to 125 base pair (bp) fragments. An additional fragmentation step further reduced the amplified products to segments of approximately 25–50 bp, which were then end-labeled using biotinylated nucleotides. Next, the samples were denatured and transferred to the hyb tray, after which we prepared the samples for begin hybridization in the GeneTitan MC Instrument (Affymetrix). The hybridization step followed the GeneTitan Multichannel Instrument User’s Manual, (P/N 08–0306), using an Axiom BiobankPlus Genotyping Array KNIHv1.0. After ligation, the arrays were stained and imaged on the GeneTitan MC Instrument (Affymetrix). The obtained images were analyzed according to the Affymetrix GeneChip Command Console Software User Manual (P/N 702569, http://www.thermofisher.com/kr/ko/home.html). Genotype data were produced using the Hybridization on Affymetrix Axiom KORV1.0-96 Array available through the K-CHIP consortium. This array was designed by the Center for Genome Science, Korea National Institute of Health, Korea (4845-301, 3000–3031, http://nih.go.kr/NIH_NEW/main.jsp). Genotyping was performed by DNA Link, Inc. BRLMM-P was the method used for genotype calling (https://media.affymetrix.com/support/developer/powertools/changelog/apt-probeset-genotype.html). Call rates for each individual are shown at Supplementary Dataset.

### Clinical and colonoscopy assessment

Each subject completed a past medical history questionnaire, and an anthropometric assessment was performed. The colonoscopy for colorectal cancer screening and surveillance was performed by board-certified gastroenterologists, who had each performed more than 2000 colonoscopies. Bowel preparation was performed with 4 L of polyethylene glycol lavage, and the effectiveness of the bowel preparation was graded according to the Aronchick Bowel Preparation Scale^15^. The cleanliness of the total bowel was scored as one of the following five grades: excellent, good, fair, poor, and inadequate.

For the diagnosis of diverticulosis, the colonoscopy reports with images of the enrolled cohort were reviewed. In cases of patients who had previously visited the center, their earlier medical records were also reviewed. The diverticulosis location was defined as follows: left sided was defined as the sigmoid colon, descending colon, and rectum, and right sided was defined as the cecum, ascending colon, and transverse colon. To identify the etiologic genetic factors affecting right-sided diverticulosis, which is mainly found in the Asian population, we established a case group of patients with right-sided or bilateral diverticulosis at any age. Additionally, we established a control group of individuals in whom no diverticulosis was detected in at least two consecutive colonoscopies performed after the age of 55 to maximize the effect of the genetic predisposition.

### Ethics statement

The Institutional Review Board of the Seoul National University Hospital approved the use of the biorepository data with informed consent (IRB number 1103-127357). We used retrospectively collected clinical and genetic data; the board approved this study protocol (IRB number 1602-084-741) and waived informed consent. The study was performed in accordance with the Declaration of Helsinki.

### Quality control and statistical analysis

We performed systematic quality control steps on the raw genotype data and obtained a total of 755,820 SNPs; SNPs with case and control minor allele frequencies <1%, case or control call rates <95% or a significant deviation from Hardy–Weinberg equilibrium in controls (P < 0.0001) were excluded. We also excluded SNPs likely to be false-positive associations due to incorrect clustering. Analysis of population stratification was performed to assess the influence of ethnicity using principal component analysis (PCA, Supplementary Fig. 1). The total population of our study was merged with YRI and CEU data from the 1000 Genomes data for PCA. Among the markers that passed the quality control criteria [minor allele frequencies >0.05, call rates >0.05, Hardy–Weinberg equilibrium (P > 0.0001), autosomal], there were 220,222 overlapping markers in the datasets. We randomly selected 20% of the overlapping markers (43,979) for PCA plotting. In the PCA plot, the Korean population showed distinct clustering. This step of the analysis was performed with the EIGENSIFT version 6.1.4 package.

Logistical regression analyses were used to calculate the odds ratios (ORs), 95% confidence intervals (CIs) and the corresponding P-values for each SNP, controlling for sex as a covariate in the additive model. Since the majority of Korean populations are ethnically homogenous^16^ and the Korean population included in our study showed a distinct clustering in the PCA plot, we did not adjust for principal component scores. Multiple testing of the associations was conducted by the Bonferroni correction criteria. SNPs that were 200 kb apart were closely related. Statistical tests were performed using PLINK version 1.9 (https://www.cog-genomics.org/plink2), SAS 9.1. SAS Institute Inc., Cary NC and R 3.2.2 (R Development Core Team; R Foundation for Statistical Computing, Vienna, Austria).

The results were verified using the test and the validation sets. We divided the enrolled population into two groups based on their time of enrollment. Samples donated between January 2014 and April 2015 composed the test set (n = 5,693), and those enrolled between May 2015 and December 2015 composed the replication set (n = 2,255). The intention was to reevaluate in the replication set any SNPs that had P-values of less than 5 × 10^−8^ in the test set. However, since no SNPs had P-values less than 5 × 10^−8^ in the test set, rather than applying Bonferroni’s correction criteria, we selected SNPs that had a less stringent P-value cutoff (1 × 10^−3^), with at least 2 SNPs aggregated within 200 kb of the location. SNPs that showed P-values less than 0.05 were considered significant in the validation set. Regional plotting was performed with the LocusZoom program (http://locuszoom.org).

## Results

### Baseline characteristics of the subjects

Among the total 7,948 enrolled subjects, 1,327 (16.7%) had colonic diverticulosis. The enrollment process and characteristics of the case and control groups are described in Fig. 1 and Table 1, respectively. According to the inclusion criteria, a total of 1,968 individuals (893 cases and 1,075 controls) and 651 individuals (305 cases and 346 controls) were included in the test and replication sets, respectively. A quantile–quantile (Q-Q) plot is shown in Supplementary Fig. 2.

**Table 1:** Clinical characteristics of the test and replication set study subjects.

	Combined set of subjects			Test set subjects		P	Replication set subject		P
	Controls (n = 1380)	Cases (n = 1239)	P	Controls (n = 1075)	Cases (n = 893)		Controls (n = 305)	Cases (n = 346)	
Male (%)	904 (65.5)	903 (72.9)	<0.001	709 (66.0)	664 (74.4)	<0.001	195 (63.9)	239 (69.1)	0.192
Age (mean ± SD)	61.5 ± 5.4	54.6 ± 8.9	<0.001	61.5 ± 5.6	55.3 ± 8.7	<0.001	61.6 ± 4.8	52.9 ± 9.4	<0.001
BMI (mean ± SD)	23.5 ± 2.7	24.0 ± 2.9	<0.001	23.4 ± 2.7	24.0 ± 2.7	<0.001	23.8 ± 2.6	24.2 ± 3.2	0.071
DM (%)	115 (8.3)	91 (7.3)	0.387	87 (8.1)	67 (7.5)	0.688	28 (9.2)	24 (6.9)	0.363
HTN (%)	418 (30.3)	338 (27.3)	0.098	322 (30)	253 (28.3)	0.461	96 (31.5)	85 (24.6)	0.061
Smokers (%)	655 (47.5)	708 (57.1)	<0.001	521 (60.2)	532 (68.2)	0.001	134 (57.5)	176 (58.9)	0.822

SD, standard deviation; BMI, body mass index; DM, diabetes; HTN, hypertension.

### Genome-wide association study for right-sided colonic diverticulosis

**Figure 2:**
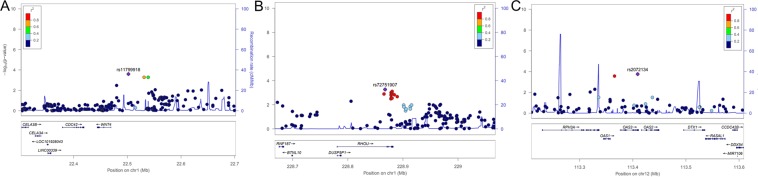
Signal plot for the significant loci in the test set of the GWAS (**A**) around the WNT4 locus on chromosome 1, (**B**) around the RHOU locus on chromosome 1, and (**C**) around the OAS1/3 loci on chromosome 12.

A sex-adjusted GWAS of 755,820 SNPs was performed using the colonoscopic findings of right-sided or bilateral colonic diverticulum. The GWAS on right-sided diverticulosis identified 9 SNPs in three SNP aggregates; three SNPs (Fig. 2A) were found on chromosome 1, located between 2250200 and 2253878 (the most significant SNP was rs11799918, P = 2.532 × 10^−4^); four additional SNPs (Fig. 2B) were also found on chromosome 1, located between 228867648 and 228880466 (the most significant SNP was rs72751907, P = 5.441 × 10^−4^); and two final SNPs (Fig. 2C) were found on chromosome 12, located between 113365621 and 113409176 (the most significant SNP was rs2072134, P = 1.750 × 10^−4^).

**Figure 3:**
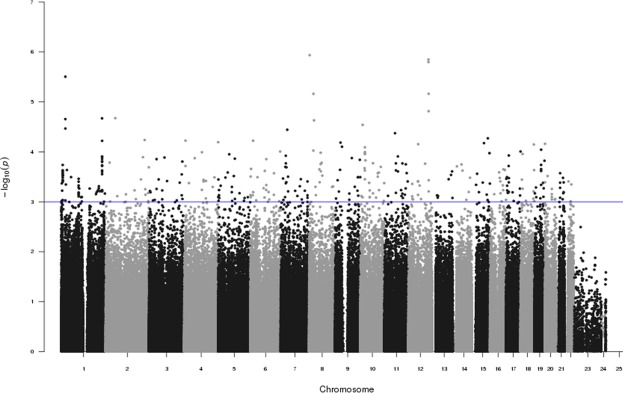
Manhattan plot of the combined set for the right-sided diverticulosis GWAS.

The 9 SNPs were genotyped in the replication set. These SNPs were validated in the replication set, with P < 0.05 (Table 2). The genotype counts for each SNP are shown in Supplementary Fig. 3. Three SNPs were in the WNT4 gene (the most significant SNP was rs2473253, OR = 1.668 [CI: 1.232–2.259], P = 9.412 × 10^−4^), four SNPs were in the RHOU gene (the most significant SNP was rs72751907, OR = 0.6407 [CI: 0.435–0.9435], P = 2.419 × 10^−2^), and two SNPs were in the 2′-5′-oligoadenylate synthetase 1, 3 (OAS1/3) genes (the most significant SNP was rs2072134, OR = 0.676 [CI: 0.57–0.8018], P = 1.033 × 10^−2^). The Manhattan plots for the combined set of SNPs are shown in Fig. 3.

**Table 2:** Logistic regression analysis results of the GWAS for right-sided colonic diverticulosis (as covariates of sex).

Chr	SNP	Position	Gene	M	Combined set			Test set			Replication set		
					MAF (case/control)	OR (L95, U95)	P	MAF (case/control)	OR (L95, U95)	P	MAF (case/control)	OR (L95, U95)	P
1	rs11799918	22502000	WNT4	A	0.183/0.139	1.39 (1.194, 1.618)	2.211 × 10^−5^	0.180**/**0.136	1.391 (1.166, 1.661)	2.532 × 10^−4^	0.190**/**0.148	1.358 (1.006, 1.834)	0.046
1	rs75637000	22531206	WNT4	T	0.190/0.146	1.366 (1.179, 1.584)	3.411 × 10^−5^	0.186**/**0.143	1.353 (1.141, 1.605)	5.241 × 10^−4^	0.198**/**0.152	1.381 (1.027, 1.856)	0.033
1	rs2473253	22538787	WNT4	T	0.195/0.145	1.415 (1.223, 1.637)	3.128 × 10^−6^	0.191**/**0.148	1.345 (1.138, 1.59)	5.246 × 10^−4^	0.206**/**0.134	1.668 (1.232, 2.259)	0.001
1	rs72751907	228867648	RHOU	T	0.065/0.096	0.657 (0.535, 0.806)	6.014 × 10^−5^	0.062**/**0.092	0.651 (0.510, 0.830)	5.441 × 10^−4^	0.071**/**0.108	0.641 (0.435,0.944)	0.024
1	rs4993975	228878669	RHOU	C	0.067/0.096	0.676 (0.552, 0.829)	1.591 × 10^−4^	0.065**/**0.056	0.668 (0.525, 0.849)	9.677 × 10^−4^	0.074**/**0.108	0.669 (0.456, 0.981)	0.039
1	rs11583565	228880135	RHOU	T	0.068/0.097	0.676 (0.552, 0.828)	1.537 × 10^−4^	0.065**/**0.093	0.667 (0.524, 0.848)	9.368 × 10^−4^	0.073**/**0.108	0.669 (0.456, 0.981)	0.039
1	rs11580020	228880466	RHOU	A	0.067/0.097	0.672 (0.548, 0.823)	1.264 × 10^−4^	0.064**/**0.093	0.662 (0.520, 0.842)	7.780 × 10^−4^	0.074**/**0.033	0.667 (0.454, 0.979)	0.038
12	rs11066453	113365621	OAS1, OAS3	G	0.111/0.150	0.6948 (0.589, 0.82)	1.535 × 10^−5^	0.109**/**0.148	0.701 (0.579, 0.849)	2.686 × 10^−4^	0.114**/**0.157	0.666 (0.479, 0.928)	0.016
12	rs2072134	113409176	OAS1, OAS3	A	0.100/0.140	0.676 (0.57, 0.802)	6.88 × 10^−6^	0.099**/**0.137	0.685 (0.562, 0.834)	1.750 × 10^−4^	0.102**/**0.148	0.641 (0.456, 0.901)	0.01

Chr, chromosome; rs number, SNP ID in dbSNP database; M, minor allele; MAF, minor allele frequency; OR, odds ratio; L95 and U95, lower and upper 95% CIs, respectively.

## Discussion

The pathogenesis of diverticulosis has long been discussed, but the cause of diverticulosis is still unclear. Diverticulosis is a disease resulting from complex interactions among the aging process, multiple environmental factors such as diet and lifestyle, and genetic predisposition^9^. Recent evolving knowledge also suggests that abnormalities in colonic motility, changes in colonic muscle morphology, chronic low-grade inflammation of the colonic wall, and connective tissu e abnormality in the colon wall are associated with colonic diverticulosis^1,9,17^. Anatomically, colonic diverticulosis develops between the taenia coli, where the vasa recta penetrates the colonic wall muscle, which is the weakest point of the colonic wall^1^. Known pathological features are thickened muscularis propria^18,19^, changes in the collagen balance in the colon wall^18^, instances of angiodysplasia^20,21^, thickened abnormal vessels^18^, and increased myenteric plexus with fewer ganglion cells^22^.

This was the first GWAS for diverticulosis. In this study, although the statistical power did not meet the Bonferroni correction criteria, the results suggest three novel candidate genes that might be associated with diverticulosis. Our results might offer important information regarding the pathogenesis of diverticulosis. The SNPs rs11799918, rs75637000, and rs2473253 are linked near the WNT4 gene (wingless-type MMTV integration site family member 4). WNT4 is known to be related to vascular smooth muscle cell proliferation^23^. There was a study that investigated the association between diverticulosis and arterial smooth muscle and showed that atherogenesis caused hypertrophy in colon muscle cells^24^. The regulatory function of WNT4 in vascular smooth muscle cell proliferation and collagen expression could suggest the role played by WNT in the mechanism underlying the development of diverticulosis^25^.

The SNPs rs72751907, rs4993975, rs11583565 and rs11580020 are linked near the RHOU gene (Ras homolog family member U, also known as = WNT1-RESPONSIVE CDC42 HOMOLOG; WRCH1). The RHOU gene is known to mediate the WNT signaling pathway, which regulates cell morphology, cytoskeletal organization and cell proliferation^26^. Like WNT4, RHOU also functions as a proangiogenic molecule and enhances human endothelial progenitor functioning^27^. One of the complications of colonic diverticulosis is diverticular bleeding, which is the most common cause of lower gastrointestinal bleeding^28^. The pathogenesis of diverticular bleeding is postulated to involve exposure of the penetrating vessel for the colonic wall, which weakens at the point of herniation, to traumatic injury, resulting in bleeding^29^. In a colon specimen from a diverticular patient, a large arterial branch arching over the dome of the diverticulum was observed^30^. Since WNT4 and RHOU exhibit functions in the proangiogenic and proliferating endothelium, these genes might underlie the pathophysiologic mechanism of diverticular bleeding.

WNT4 and RHOU are both associated with the WNT family. WNT family proteins are reported to play important roles in the development of the gut^31,32^ and the homeostasis of the intestine epithelium^33^. There was a study performed in rats that showed that WNT gene signaling is involved in intestinal neuronal and glial differentiation and that under inflammatory stimulation, WNT signaling results in anti-inflammatory activity in the enteric nervous system^34^. Based on these reports, we suggest that WNT family genes play a pivotal role in the development of right-sided colonic diverticulosis, especially in early life stages.

Rs11066453 and rs2072134 were linked near the OAS1/3 genes. The OAS family of proteins is induced by interferon and is associated with the antiviral and apoptotic responses^35^. The level of interferon is known to play a pivotal role in host protection and immunopathology in response to mucosal pathogens and during inflammation in the gut^36^. In a study in rat colons, cytotoxic insult to the colon mucosa induced increased OAS1 gene expression^37^. According to that study, the OAS gene locus could be related to chronic low-grade inflammation of the colonic wall, which is thought to be the pathophysiology underlying the development of diverticulosis.

The major characteristics of this study are as follows. First, most diverticulosis is asymptomatic, and only 20% of patients manifest complicated symptoms^38^. Therefore, it is difficult to determine the actual prevalence of diverticulosis and to carry out a genetic study on colonic diverticulosis that includes the asymptomatic population, which may be why there have been no genetic studies conducted on colonic diverticulosis. Fortunately, in Korea, where self-paid health check-ups are widely performed, colonoscopy is recommended from the age of 50 on for colorectal cancer screening. Therefore, it was possible for us to detect diverticulosis in a healthy population and to perform a GWAS. Second, we investigated the genetic risk factors in a Korean population for right-sided diverticulosis, which is thought to be true diverticula, including all the layers of the colon. This type of diverticulosis is completely different from left-sided diverticulosis, which has been considered false diverticula in a Western population. Epidemiologic studies show that right-sided diverticulosis is developed at an earlier age and, unlike left-sided diverticulosis, is thought to be congenital^39–42^. Therefore, the strong genetic association with the development of right-sided diverticulosis allowed us to identify genes involved in the susceptibility to right-sided diverticulosis by a GWAS. Third, we used the Affymetrix Axiom KORV1.0-96 Array, available through the K-CHIP consortium. The characteristics of the array have been described elsewhere^43^. Briefly, it contains approximately 830,000 SNPs, including functional SNPs such as nonsynonymous SNPs, HLA region variants, eQTLs, and previously reported disease-associated SNPs; shows 99.77% reproducibility and 99.73% accuracy; and exhibits imputation-based genomic coverage^44^ of common variants (minor allele frequency >5%) is over 95%.

This study has several limitations. The diagnosis of diverticulosis in this study was determined solely by colonoscopy. Therefore, technical issues could lead to missing cases of diverticulosis. To overcome this limitation, we included a control population of individuals who had negative findings in at least 2 complete colonoscopies after proper bowel cleansing. Second, no SNPs passed the Bonferroni correction criteria. As a result, we cannot conclusively describe the association between the novel SNPs and diverticulosis. To compensate for this limitation, we used a less strict P-value cutoff for SNPs that were aggregated within 200 kb. Additionally, we performed a joint analysis, consisting of a GWAS for the combined test and replication sets. One study has shown that jointly analyzing the data from test and replication sets results in increased power to detect genetic associations^45^. In the combined set, rs11799918, rs2473253, rs11066453 and rs2072134 exhibited P values of less than 5 × 10^−5^. The power of significance was stronger than that in the two-stage GWAS but still did not meet the Bonferroni correction criteria. This limitation should be addressed in future studies with larger sample sizes. Third, since the focus of this study was on right-sided diverticulosis in a Korean population, this study result may not apply to individuals of Western populations, who predominantly suffer from left-sided diverticulosis. However, the results of this study could explain the pathogenesis of right-sided diverticulosis in Mongolian people from many Asian countries including Japan, Korean, and China. Fourth, there can be several grades of right-sided diverticulosis, ranging from single to multiple diverticuli. However, we could not obtain statistical significance related to multiple right-sided diverticulosis (data not shown). This findings suggests that our study may not show statistical significant to clinically severe right-sided case. Since the sample size was not insufficient for multiplicity analysis, it should be performed in a larger population set.

In summary, we report the first GWAS of colonic diverticulosis and suggest possible candidate genes that might explain the pathophysiology of right-sided colonic diverticulosis. The genetic mechanism related to the WNT and OAS genes might be the underlying cause of the development of right-sided diverticulosis. Our findings could be the cornerstone for further genetic investigation of colonic diverticulosis and provide important information for the development of new treatment options and prevention strategies for diverticular disease. Since this study was limited to a relatively small number of individuals of Korean ethnicity, further studies are needed to replicate the results in a larger sample size.

## Supplementary information

Call rates for each individual

Supplementary figures
