# Supplementary material for: Genome-wide association study of right-sided colonic diverticulosis in a Korean population
Source: Sci Rep. 2019 May 14;9:7360. doi: 10.1038/s41598-019-43692-8 (PMC6517584; doi:10.1038/s41598-019-43692-8)
Supplement: Supplementary file 2 — Supplementary figures [file 41598_2019_43692_MOESM2_ESM.pdf]

Genome-wide association study of right-sided colonic diverticulosis in a Korean population

Eun Kyung Choe, Jong-Eun Lee, Su Jin Chung, Sun Young Yang, Young Sun Kim, Eun-Soon Shin, Seung Ho Choi, Jung Ho Bae

Supplementary Figure Legends

Supplementary Figure 1. Principal component analysis (PCA) for the assessment of population stratification. The total population of our study merged with YRI and CEU data from the 1000 Genomes Project for PCA. Among the markers that passed the quality control criteria [minor allele frequencies > 0.05, call rates > 0.05, Hardy–Weinberg equilibrium (P > 0.0001), autosome], there were 220,222 overlapping markers in the datasets. We randomly selected 20% of the overlapping markers (43,979) for PCA plotting.

Supplementary Figure 2. Q-Q plot of the right-sided diverticulosis GWAS.

Supplementary Figure 3. Genotype counts for each SNPs

Figure S1

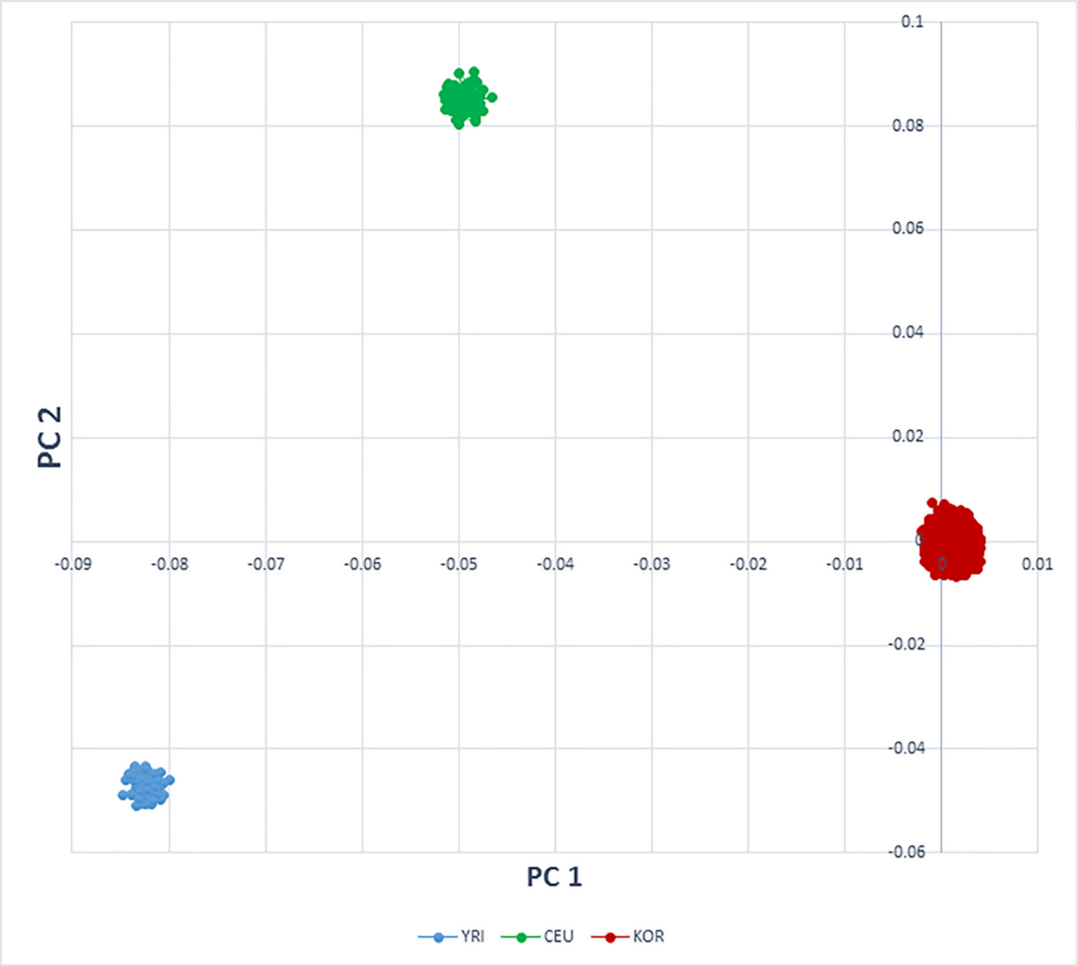

Figure S2

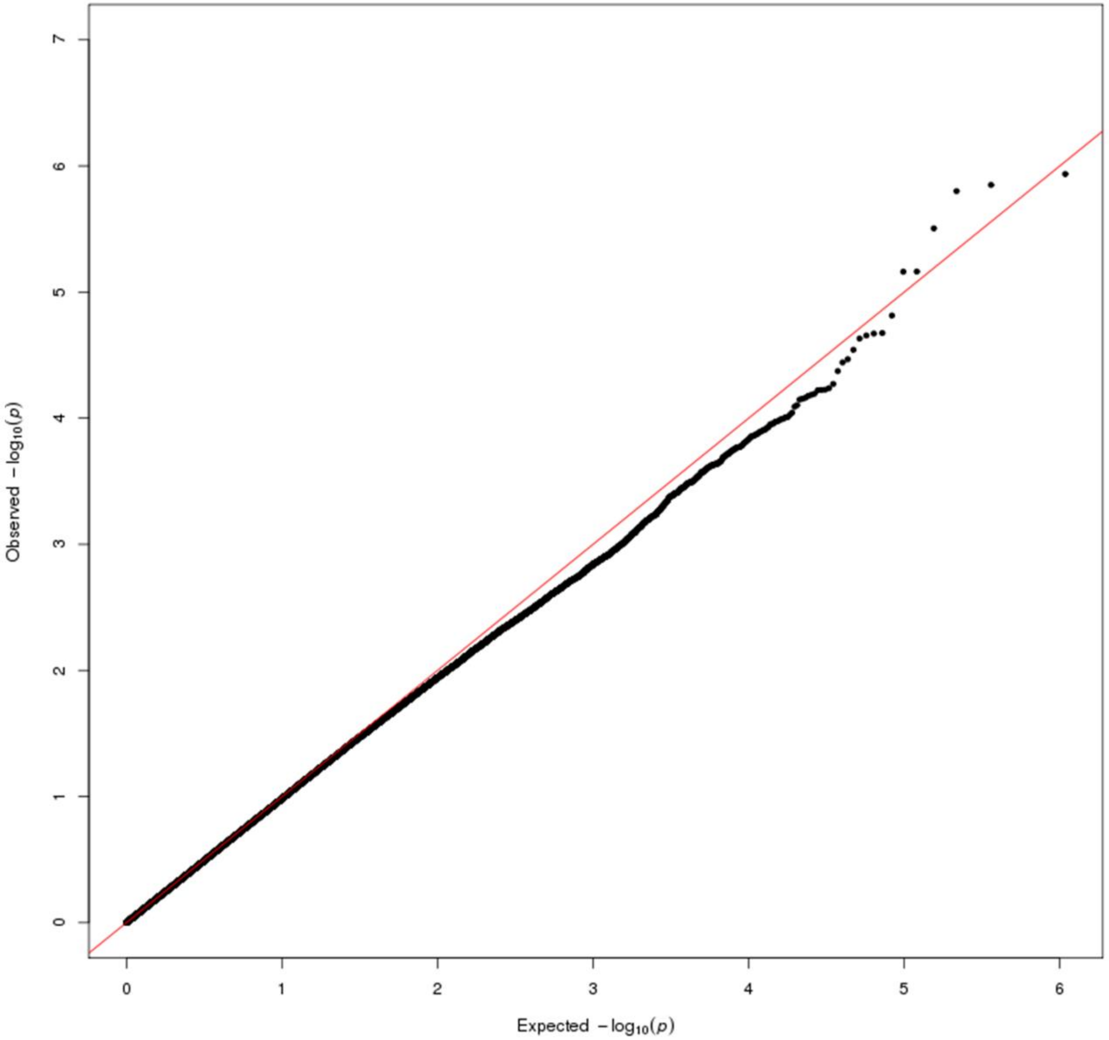

Figure S3

SNP	Group	Test set			Replication set		
		GG	GA	AA	GG	GA	AA
rs11799918	Case	597	265	28	223	106	12
	Control	788	265	13	215	78	5
		GG	GT	TT	GG	GT	TT
rs75637000	Case	594	265	34	222	111	13
	Control	783	270	19	216	85	4
		CC	CT	TT	CC	CT	TT
rs2473253	Case	585	273	34	218	112	15
	Control	783	263	27	226	76	3
		CC	CT	TT	CC	CT	TT
rs72751907	Case	783	104	4	298	47	1
	Control	884	184	7	244	56	5
		AA	AC	CC	AA	AC	CC
rs4993975	Case	782	106	5	295	49	1
	Control	881	186	7	244	56	5
		CC	CT	TT	CC	CT	TT
rs11583565	Case	781	106	5	295	49	1
	Control	879	186	7	244	56	5
		GG	GA	AA	GG	GA	AA
rs11580020	Case	782	105	5	295	49	1
	Control	880	186	7	243	56	5
		AA	AG	GG	AA	AG	GG
rs11066453	Case	711	168	14	272	69	5
	Control	778	274	22	212	90	3
		GG	GA	AA	GG	GA	AA
rs2072134	Case	728	153	12	279	61	5
	Control	799	253	21	217	84	3
